# Testosterone Treatment and Sexual Function in Men: Secondary Analysis of the T4DM (Testosterone for Diabetes) Trial

**DOI:** 10.1210/clinem/dgaf060

**Published:** 2025-02-10

**Authors:** Gary A Wittert, Kristy P Robledo, David J Handelsman, Warrick J Inder, Bronwyn G A Stuckey, Bu B Yeap, Karen Bracken, Carolyn A Allan, David Jesudason, Alicia Jenkins, Andrzej S Januszewski, Mathis Grossmann

**Affiliations:** Freemasons Centre for Male Health and Wellbeing, University of Adelaide, Adelaide, SA 5000, Australia; Department of Endocrinology, The Queen Elizabeth Hospital, Woodville, Adelaide, SA 5011, Australia; NHMRC Clinical Trials Centre, University of Sydney, Sydney, NSW 2050, Australia; ANZAC Research Institute, University of Sydney and Department of Andrology, Concord Hospital, Sydney, NSW 2139, Australia; Department of Endocrinology, Princess Alexandra Hospital and the University of Queensland, Brisbane, QLD 4102, Australia; Keogh Institute for Medical Research, Department of Endocrinology and Diabetes, Sir Charles Gairdner Hospital and Medical School, University of Western Australia, Nedlands, WA 6009, Australia; Medical School, University of Western Australia and Department of Endocrinology and Diabetes, Fiona Stanley Hospital, Perth, WA 6150, Australia; Sydney Musculoskeletal Health, Faculty of Medicine and Health, The University of Sydney, Camperdown, NSW 2050, Australia; Centre for Endocrinology and Metabolism, Hudson Institute of Medical Research, School of Clinical Sciences at Monash Health, and Faculty of Medicine, Nursing and Health Sciences, Monash University, Clayton, VIC 3168, Australia; Freemasons Centre for Male Health and Wellbeing, University of Adelaide, Adelaide, SA 5000, Australia; Department of Endocrinology, The Queen Elizabeth Hospital, Woodville, Adelaide, SA 5011, Australia; Diabetes and Vascular Medicine, Baker Heart and Diabetes Institute, Melbourne, Melbourne, VIC 3074, Australia; Sydney School of Pharmacy, Faculty of Medicine and Health, The University of Sydney, Canperdown, NSW 2050, Australia; Department of Medicine and Endocrinology, Austin Health, and University of Melbourne, Heidelberg, VIC 3079, Australia

**Keywords:** testosterone, sexual function, diabetes, obesity, clinical trial

## Abstract

**Context:**

The combined effects of testosterone treatment and lifestyle intervention on sexual function in men at high risk of type 2 diabetes are unclear.

**Objective:**

To assess the effect of testosterone treatment with a lifestyle intervention in men aged 50 to 74 years at high risk of, or newly diagnosed with, type 2 diabetes (via oral glucose tolerance test).

**Design:**

A secondary analysis of the Testosterone for the Prevention of Type 2 Diabetes trial, a double-blind, placebo-controlled trial conducted across 6 Australian centers.

**Interventions:**

Intramuscular testosterone undecanoate (1000 mg) or placebo, 3 monthly for 2 years alongside a community-based lifestyle program.

**Main outcomes:**

Sexual function measured using the International Index of Erectile Function (IIEF)-15 questionnaire.

**Results:**

Of 1007 participants, 792 (79%) had complete International Index of Erectile Function-15 data. Baseline domain scores were inversely related to age and waist circumference, but unrelated to serum testosterone or estradiol levels. Testosterone treatment improved all 5 International Index of Erectile Function-15 domain scores, with stronger effects on sexual desire and orgasmic function in older men, and sexual desire in men with higher depression scores. Testosterone had no impact on depression. Independent of treatment, reductions in waist circumference were associated with improved erectile function, and reductions in depression scores correlated with better sexual function. Clinically significant improvement in erectile function and sexual desire occurred in 3% and 10% of men, respectively, and was inversely related to baseline function. Clinically significant improvement improvements in erectile function and sexual desire were greater in younger and older men respectively.

**Conclusion:**

Testosterone treatment enhanced sexual desire and, to a lesser extent, erectile function, particularly in older men and those with higher waist circumference or depressive symptoms. Reduced waist circumference and depression independently improved sexual function.

Testosterone and its aromatized metabolite estradiol are important for optimal sexual function in men (1). The association of sexual effects with serum testosterone concentration varies and is influenced by age, psychosocial, lifestyle behaviors, and the presence of, or risk factors for, chronic disease (2, 3). Sexual desire is the symptom most closely related to serum testosterone concentration while also influenced by psychosocial factors and chronic disease, for example depression, and obstructive sleep apnea. Erectile function, by contrast as a hydraulic neurovascular function, has a much weaker association with serum testosterone concentration but is strongly associated with cardiometabolic and other chronic vascular disorders (2, 3).

In young men with hypogonadism resulting from pathology affecting the hypothalamic-pituitary-testicular axis, treatment with testosterone restores sexual function (4). In observational studies, obesity, cardiometabolic disease, depression, sexual dysfunction, and lowered serum testosterone concentrations are associated (5, 6), but the apparent age-related decrease of serum testosterone concentrations is primarily the consequence of obesity and accumulated obesity-related comorbidities and depression (7). By design, such observational studies cannot ascribe a causal relationship between the lowered serum testosterone and sexual dysfunction, and it is plausible or likely that obesity and metabolic syndrome may cause both sexual dysfunction and a lowered serum testosterone concentration.

In older men with preexisting cardiovascular disease and serum testosterone <300 ng/dL (10.4 nmol/L) participating in TRAVERSE, testosterone treatment compared with placebo improved sexual desire (Hypogonadism Impact of Symptoms Questionnaire libido subdomain score) (8) by 3.9 points and activity by 3.3 events per week but not erectile function on the IIEF. In the T-Trials, which recruited older men with serum testosterone <275 ng/dL (9.5 nmol/L) not preselected for vascular disease, testosterone treatment improved sexual desire (Derogatis Interview for Sexual Function Sexual Desire Domain) (9) by 2.93 points and erectile function (IIEF) by 2.64 points (10).

By contrast, studies have reported that lifestyle measures including exercise and diet to achieve weight loss (11, 12) can significantly improve sexual desire and erectile function and increase serum testosterone concentrations (3 ± 7.7 nmol/L; 86.6 ± 222 ng/dL). The average increase in score on the erectile function subscale on the IIEF was 3 points (maximum 30) with a 10% or greater weight loss with diet and physical activity (11) or 2.2 points with 10% or greater weight loss with diet alone (12).

However, to date, the combined effects of treatment with testosterone and lifestyle intervention have not been studied in men without pathologic hypogonadism. To address this clinically important issue, we took advantage of the unique design of the Testosterone for the Prevention of Type 2 Diabetes (T4DM) study, which randomized men at high risk of or with newly diagnosed type 2 diabetes to either testosterone or placebo on the background of a WW (formerly Weight Watchers)-directed lifestyle program administered to all randomized men. We have previously reported that in T4DM testosterone treatment, relative to placebo on the background of the WW lifestyle program, improved sexual desire and erectile dysfunction to a small but statistically significant degree (13). In the current analysis, we aimed to determine, in relation to effects of a lifestyle intervention and testosterone treatment on sexual function, baseline predictors of response, interactions with changes in waist circumference, serum glucose, sex steroid concentrations, and mood, and to identify predictors of clinically significant benefits.

## Methods

### Data Source

The T4DM protocol (14) and efficacy results (13) have been published. Briefly, T4DM was a multicenter Australian randomized, double-blind, placebo-controlled, 2-year, phase 3b trial designed to evaluate the effects of testosterone treatment, on the background of a lifestyle intervention program on diabetes risk, as assessed by 2 coprimary outcomes: (1) 2-hour glucose ≥11.1 mmol/L (200 mg/dL) and (2) the change in 2-hour glucose from baseline, both as measured by the oral glucose tolerance test (OGTT) at 2 years. To be eligible, participants had to be male, aged 50 to 74 years, with a waist circumference ≥95 cm, have impaired glucose tolerance or newly diagnosed type 2 diabetes, baseline Center for Epidemiological Studies-Depression (CES-D) score <16, and a screening fasting serum testosterone drawn between 8 and 10 Am of ≤14 nmol/L (403 ng/dL) by immunoassay at an accredited pathology provider (Sonic Health Care, Australia). All on-study testosterone assays were performed by liquid chromatography mass spectrometry (LCMS) at trial completion. Exclusion criteria included organic hypothalamic-pituitary-testicular pathology, testosterone treatment in the past 12 months, psychiatric disorder, chronic viral infection, stroke or transient ischemic attack in previous 3 years, major cardiovascular event in previous 6 months, cardiac failure, angina, arrhythmias, blood pressure of 160/100 mm Hg or higher, family history of thrombophilia, hematocrit of 50% or more, current or previous malignancy (excluding nonmelanoma skin cancer), abnormal liver or renal function, previous or planned bariatric surgery, use of antiobesity drugs, or history of substance abuse at any time. All 1007 participants were given access to the WW lifestyle program, which provided an interactive website and weekly group meetings; participants were encouraged to engage in both. The interactive website provided diet and activity guidelines, and self-monitoring tools that allowed men to log food, physical activity, and weigh-in details. Participants were randomized (1:1) to testosterone undecanoate (1000 mg) or matched placebo, both administered by clinic staff via deep intramuscular injection every 3 months for 2 years. Discontinuation from treatment before 2 years was 26% (131/503) in placebo and 23% (116/504) in testosterone groups, mostly because of participant preference (79% vs 56%, 104/131 vs 65/116) or a protocol-specified rise in hematocrit (1% vs 22%; 1/131 vs 25/116) in the placebo and testosterone groups, respectively. The study received ethics committee approval to be conducted at each site and was registered on the Australia and New Zealand Clinical Trials Registry (ACTRN12612000287831).

#### Questionnaires

Sexual function was assessed using the validated International Index of Erectile Function (IIEF-15)—a 15-item questionnaire with 5 individual domains. Each question scored between 0 and 5, for a maximum score of 75, with higher scores indicating better sexual function. The 5 domains with maximum scores in brackets are erectile function (15), orgasmic function (10), sexual desire (10), intercourse satisfaction (16), and overall satisfaction (10, 16).

Depressive symptoms were assessed with the CES-D questionnaire (17). The CES-D is a 20-item measure that asks individuals to rate how often over the past week they experienced depressive symptomology, such as restless sleep, poor appetite, and feeling lonely. Response options range from 0 to 3 for each item (0 = rarely or none of the time, 1 = some or little of the time, 2 = moderately or much of the time, 3 = most or almost all the time). Scores range from 0 to 60, with high scores indicating greater depressive symptoms.

T4DM trial participants completed the IEFF-15 questionnaire at baseline, and weeks 30, 54, 78, and 104 (study end). The CES-D questionnaire was completed at baseline, week 54, and week 104.

#### Statistical methods

The secondary, prespecified analyses were performed by intention to treat, using all available data up until the completion of the trial at 2 years. Baseline characteristics were compared for participants with “complete data” (ie, all 5 IIEF-15 domains at all timepoints are available) vs those without, using 2-sample tests or chi-squared tests as appropriate. Subsequent analyses included all available data over time for each subscale. First, baseline associations between IIEF-15 domains were assessed with baseline characteristics using linear regression. Effects in those with baseline T (<12 nmol/L vs ≥12 nmol/L) with baseline risk factors were also assessed by fitting interactions.

Next, IIEF-15 domains were assessed to determine if changes over time were consistent between treatment arms. Each domain was modeled over time using a linear mixed-effects model, adjusting for visit, treatment, and the relevant baseline domain, while accounting for the correlation within each subject over time (random effect), as each domain was assessed at 4 timepoints on-study. An interaction term was fit to assess if there the trends over time varied by treatment group (treatment by visit interaction). If the interaction was *P* > .05, the interaction was dropped from subsequent models.

The effect of testosterone was then estimated using linear mixed-effects models for each subscale, adjusted for these covariates as well as the following baseline risk factors: the ratio of serum testosterone and estradiol (T:E2) concentrations (by LCMS), age, OGTT 2-hour serum glucose, CES-D score, marital status, education level, occupational status, smoking status, study site, and waist circumference. Interactions between these baseline risk factors, as well as baseline T < 12 nmol/L vs T ≥ 12 nmol/L (as requested by a reviewer), and treatment were assessed. The ratio of serum T:E2 ratio (by LCMS) was used because serum testosterone and estradiol have been shown to better predict treatment response in older hypogonadal men than testosterone alone (18) and it provided a better statistic fit (ie, Akaike information criterion an indicator of model fit) than if they were both included in the model together.

Sensitivity analyses additionally explored the impact of selective serotonin reuptake inhibitors (SSRIs) and phosphodiesterase type 5 inhibitor (PDE5-I) on the treatment effect.

These models were then extended to allow for changes over time in waist circumference, blood pressure (both systolic and diastolic), and depression (CES-D). Changes in sex steroids over time could not be assessed because they were collected at concurrent timepoints as sexual functioning questionnaires. If any changes in effects were found, interactions between these changes and treatment were explored. An additional regression analysis for CES-D at 2 years assessed the association with testosterone treatment, adjusted for baseline CES-D and baseline serum testosterone.

Because erectile function and sexual desire are the most reported and clinically used measures of sexual function, exploratory analyses were conducted for the IEFF-15 sexual desire and erectile function domains to quantify the change into a clinically meaningful context. Improvements, of 4 points on the erectile function domain score have previously been shown to be clinically meaningful (19). For sexual desire, there is no validated increment for clinically significant improvement in the Sexual Desire Domain of the IIEF (range 0-10). We used 2 approaches to set this at a 2 or more points increment from baseline. The first was an arbitrary decision based on a mathematical equivalence to a 6-point change in the erectile function domain score (range 0-30) The second method, also indicating a 2-point increase, was extrapolated from a validated increment of the score from the single sexual desire question on the Psychosexual Daily Questionnaire (20) (described fully in supplementary eMethods, page 1 (21)): lesser changes (0-2 for sexual desire and 0-4 for erectile function) were considered as a “stable/small improvement,” and any change that was negative was considered a “deterioration.”

Logistic regression analyses were performed to identify both clinically significant increases (yes vs no) and deteriorations (yes vs no) for sexual desire and erectile function separately, adjusted for baseline sexual desire or erectile function, treatment, and the following baseline covariates of interest (based on *P* < .10 in the continuous multivariate models): age, CES-D score, and waist circumference. In separate models, changes in CES-D scores and waist circumference at 2 years were investigated. Any interactions identified between baseline risk factors in the continuous outcome models were also incorporated.

No adjustments have been made for multiple comparisons. In our statistical analysis plan for the T4DM trial, we prespecified that we would not adjust for multiple comparisons. Instead, we adopt the Schulz and Grimes approach (22) reporting all analyses performed within each paper (transparency) and interpreting the totality of the results of these analyses (internal consistency). *P* values < .05 were considered statistically significant. All analyses were performed in R version (4.1.3) (23) using R packages gtsummary (24), lme4 (25), purrr (26), and tidyverse (27).

## Results

Of the 1007 participants randomized into T4DM, 932 (92% of 1007) had at least 1 IIEF-15 domain measured on study and were included in these analyses (Supplementary Table S1, Supplementary Fig. S1 (21)). A total of 74% (688/932) had complete data, whereas the remaining 26% were missing at least 1 measurement of the 5 domains over the 4 on study timepoints. Of the 932, 471 (51%) were randomized to testosterone treatment and 461 (49%) to placebo, and were well matched for baseline characteristics, including use of PDE5-Is (Table 1). Those with complete data available and those with partially or fully missing data were generally well matched in terms of baseline characteristics; however, those with data were more likely to have been randomized to treatment with testosterone, be slightly older, and have slightly lower weight with smaller waist circumferences (Supplementary Table S2 (21)). Median baseline testosterone by LCMS at baseline was 13.3 nmol/L (range 4.0-33.1) compared with 10.1 nmol/L (range 3.1-14.1) by immunoassay at screening.

**Table 1.** Baseline characteristics of those included in analyses (n = 932) by treatment

Characteristic	Placebo, N = 461	Testosterone, N = 471	*P* value*^a^*
Age at baseline (y), mean (SD)	60 (6)	60 (6)	.83
Aged 60 y or older, n (%)	223 (48)	222 (47)	.75
Current smoker, n (%)	21 (4.6)	24 (5.1)	.81
Unknown	0	1	
Waist circumference (cm), mean (SD)	118 (11)	118 (12)	.52
Waist circumference groups, n (%)			.92
≤100 cm	24 (5.2)	22 (4.7)	
101-115 cm	181 (39)	188 (40)	
>115 cm	256 (56)	261 (55)	
Weight (kg), mean (SD)	108 (17)	108 (17)	.86
BMI categories, n (%)			.55
Normal (18.50-24.99 kg/m^2^)	3 (0.7)	6 (1.3)	
Overweight (25.00-29.99 kg/m^2^)	90 (20)	76 (16)	
Obese (30.00-34.99 kg/m^2^)	169 (37)	187 (40)	
Severely obese (35.00-39.99 kg/m^2^)	133 (29)	137 (29)	
Very severely obese (≥40 kg/m^2^)	66 (14)	65 (14)	
History of T2D, n (%)	180 (39)	194 (41)	.55
History of prostate cancer, n (%)	53 (12)	53 (11)	.98
Unknown	1	0	
SSRI use at baseline, n (%)	27 (5.9)	34 (7.2)	.48
Baseline SHBG (nmol/L), mean (SD)	38 (14)	37 (14)	.82
Unknown	24	21	
Baseline LH (IU/L), mean (SD)	5.40 (2.64)	5.36 (2.82)	.43
Unknown	25	23	
Baseline FSH (IU/L), mean (SD)	7.1 (5.4)	7.1 (5.5)	.61
Unknown	25	23	
Baseline E1, mean (SD)	139 (65)	138 (61)	.97
Unknown	24	21	
Baseline E2 (pmol/L), mean (SD)	202 (103)	202 (99)	.73
Unknown	24	21	
Baseline testosterone (nmol/L), mean (SD)	13.9 (4.6)	13.4 (4.1)	.10
Unknown	24	21	
Baseline testosterone groups, n (%)			.015
Low (<8.0 nmol/L)	38 (8.7)	26 (5.8)	
Medium (8.0-<11.0 nmol/L)	82 (19)	117 (26)	
High (≥11.0 nmol/L)	317 (73)	307 (68)	
Unknown	24	21	
Baseline glucose groups, n (%)			.46
Normal (<7.8 mmol/L)*^b^*	3 (0.7)	7 (1.5)	
Prediabetes (≥7.8-<11.1 mmol/L)	365 (79)	369 (78)	
T2D (≥11.1-<15 mmol/L)	93 (20)	95 (20)	
Country of birth, n (%)			.86
Asia	12 (2.6)	12 (2.6)	
Australia/New Zealand	332 (72)	333 (71)	
Europe	72 (16)	71 (15)	
Other	45 (9.8)	54 (11)	
Unknown	0	1	
Fasting plasma glucose, mean (SD)	6.04 (0.84)	6.07 (0.89)	.68
CES-D score, mean (SD)	5.0 (4.5)	5.4 (4.6)	.19
Unknown	40	39	
Married, n (%)	394 (85)	405 (86)	.83
Unknown	0	1	
Annual income, n (%)			.38
$100 001-$150 000	94 (21)	118 (26)	
$50 000-$100 000	150 (34)	139 (31)	
<$50 000	87 (20)	84 (19)	
>$150 001	110 (25)	110 (24)	
Unknown	20	20	
Employment, n (%)			.73
Employed for wages	244 (53)	238 (51)	
Retired/unemployed	131 (28)	144 (31)	
Self-employed/family business	86 (19)	89 (19)	
Education level, n (%)			.27
Diploma	104 (23)	99 (21)	
School certificate or lower	112 (24)	122 (26)	
Trade	94 (21)	76 (16)	
University or higher	148 (32)	170 (36)	
Unknown	3	4	
Baseline PDE5 use, n (%)	14 (3.0)	13 (2.8)	.95
Baseline hypertensive use, n (%)	226 (49%)	225 (48%)	.70

Abbreviations: BMI, body mass index; CES-D, Center for Epidemiological Studies-Depression; PDE5, phosphodiesterase type 5; SSRI, selective serotonin reuptake inhibitor; T2D, type 2 diabetes.

^*a*^One-way ANOVA; Pearson's chi-squared test.

^*b*^Ten participants were enrolled on the study with a screening 2-hour glucose of 7.7 mmol/L after being granted a waiver.

### Baseline Sexual Function Effects

Age was negatively associated with all baseline sexual function scores, except for overall satisfaction (Supplementary Table S3 (21)), and was consistent over baseline testosterone groups (Supplementary Table S4 (21)). Waist circumference was negatively associated with baseline erectile function, intercourse satisfaction, and overall satisfaction, and different effects were seen by baseline testosterone groups for desire, erectile, and intercourse satisfaction, where associations were only seen in those with baseline testosterone <12 nmol/L (Supplementary Tables S5 and S6 (21)). Baseline CES-D was negatively associated with all sexual function scores, with differential effects by baseline testosterone groups for sexual desire, with effects only seen in those with baseline testosterone ≥12 nmol/L.

### On Study Sexual Function Effects

Relationships between on-study sexual function domains and baseline characteristics are given in Table 2. Average scores for all domains decreased with increasing age (*P* < .001). Sexual desire scores increased on average by 0.02 units with each 1-unit increase in CES-D score (95% CI, 0.00-0.05; *P* = .029). Erectile function scores decreased on average by 0.04 units with each 1 cm increase in waist circumference (95% CI, 0.00-0.07, *P* = .041). There was no relationship between any sexual function domain and T or E2 (all *P* > .15), or the ratio of the 2 (T:E2 ratio, all *P* > .29). T:E2 ratio was found to have a better model fit and was used for all models onward (Supplementary Table S7 (21)).

**Table 2.** Adjusted models for each IIEF domain

	Erectile function		Sexual desire		Orgasmic function		Intercourse satisfaction		Overall satisfaction	
Characteristic	Beta (95% CI)	*P* value	Beta (95% CI)	*P* value	Beta (95% CI)	*P* value	Beta (95% CI)	*P* value	Beta (95% CI)	*P* value
Randomized treatment		<.001		<.001		<.001		<.001		<.001
Placebo	—		—		—		—		—	
Testosterone	2.5 (1.7-3.4)		0.68 (0.49-0.86)		0.87 (0.52-1.2)		1.5 (1.1-1.9)		0.70 (0.46-0.95)	
Baseline domain score	0.70 (0.66-0.74)	<.001	0.61 (0.56-0.65)	<.001	0.55 (0.50-0.59)	<.001	0.69 (0.65-0.74)	<.001	0.62 (0.57-0.67)	<.001
Month of visit		.13		.007		.048		.029		.62
7	—		—		—		—		—	
13	−0.21 (−0.75 to 0.33)		−0.03 (−0.14 to 0.08)		−0.17 (−0.41 to 0.06)		−0.06 (−0.33 to 0.21)		0.02 (−0.13-to 0.17)	
18	−0.31 (−0.86 to 0.24)		−0.12 (−0.23 to 0.00)		−0.28 (−0.52 to −0.04)		−0.27 (−0.56 to 0.01)		−0.06 (−0.21 to 0.10)	
24	−0.65 (−1.2 to −0.10)		−0.18 (−0.30 to −0.07)		−0.30 (−0.54 to −0.07)		−0.37 (−0.65 to −0.09)		−0.07 (−0.22 to 0.09)	
T:E2 ratio*^a^*	1.8 (−3.5 to 7.1)	.51	−0.01 (−1.2 to 1.2)	.99	0.33 (−1.9 to 2.6)	.77	0.62 (−2.1 to 3.3)	.65	0.84 (−0.71 to 2.4)	.29
Age, y	−0.16 (−0.24 to −0.08)	<.001	−0.03 (−0.05 to −0.02)	<0.001	−0.07 (−0.10 to −0.04)	<0.001	−0.06 (−0.10 to −0.02)	.006	−0.04 (−0.06 to −0.02)	<.001
2-h glucose (OGTT)	0.17 (−0.06 to 0.41)	.15	−0.01 (−0.07 to 0.04)	.62	0.02 (−0.08 to 0.12)	.72	0.06 (−0.06 to 0.18)	.31	−0.01 (−0.08 to 0.06)	.83
CES-D score	−0.05 (−0.14 to 0.05)	.37	0.02 (0.00-0.05)	.029	−0.02 (−0.06 to 0.02)	.34	−0.01 (−0.06 to 0.04)	.68	−0.02 (−0.05 to 0.01)	.28
Marital status		.11		.53		.69		.072		.001
No long-term partner	—		—		—		—		—	
Married/de facto	1.0 (−0.24 to 2.3)		−0.09 (−0.37 to 0.19)		−0.11 (−0.64 to 0.42)		0.59 (−0.05 to 1.2)		0.63 (0.25-1.0)	
Employment		.48		.98		.44		.45		.88
Employed for wages	—		—		—		—		—	
Retired/unemployed	−0.24 (−1.4 to 0.91)		−0.01 (−0.27 to 0.25)		−0.25 (−0.74 to 0.23)		−0.28 (−0.87 to 0.31)		0.03 (−0.31 to 0.36)	
Self-employed/family business	0.54 (−0.60 to 1.7)		−0.03 (−0.28 to 0.23)		−0.26 (−0.74 to 0.22)		0.15 (−0.43 to 0.73)		0.09 (−0.25 to 0.42)	
Education		.37		.31		.45		.71		.52
Diploma	—		—		—		—		—	
School certificate or lower	0.61 (−0.62 to 1.8)		−0.05 (−0.33 to 0.22)		−0.19 (−0.70 to 0.33)		0.10 (−0.53 to 0.72)		0.17 (−0.19 to 0.52)	
Trade	0.87 (−0.49 to 2.2)		−0.17 (−0.47 to 0.14)		0.07 (−0.51 to 0.64)		−0.03 (−0.73 to 0.66)		0.16 (−0.23 to 0.56)	
University or higher	−0.08 (−1.2 to 1.1)		−0.22 (−0.47 to 0.03)		−0.31 (−0.79 to 0.17)		−0.23 (−0.81 to 0.35)		−0.05 (−0.37 to 0.28)	
Current smoker	0.00 (−2.0 to 2.0)	>.99	0.15 (−0.30 to 0.60)	.52	0.25 (−0.59 to 1.1)	.56	−0.03 (−1.0 to 0.99)	.95	−0.06 (−0.66 to 0.53)	.83
Waist circumference	−0.04 (−0.07 to 0.00)	.041	0.00 (−0.01 to 0.01)	.52	−0.01 (−0.03 to 0.00)	.063	−0.02 (−0.03 to 0.00)	.078	0.00 (−0.02 to 0.01)	.36
Site		.11		.50		.036		.15		.51
61219	—		—		—		—		—	
61319	−1.8 (−3.1 to −0.50)		−0.14 (−0.43 to 0.14)		−0.84 (−1.4 to −0.30)		−0.75 (−1.4 to −0.10)		−0.20 (−0.57 to 0.17)	
61352	−2.5 (−8.7 to 3.7)		0.80 (−0.57 to 2.2)		−0.68 (−3.3 to 1.9)		−1.4 (−4.6 to 1.7)		−0.90 (−2.7 to 0.89)	
61402	0.22 (−1.2 to 1.6)		0.17 (−0.15 to 0.50)		0.21 (−0.39 to 0.82)		0.33 (−0.39 to 1.1)		0.09 (−0.33 to 0.51)	
61512	−0.30 (−1.7 to 1.1)		−0.14 (−0.45 to 0.17)		−0.36 (−0.95 to 0.23)		0.04 (−0.67 to 0.75)		−0.01 (−0.41 to 0.40)	
61604	−0.40 (−2.9 to 2.1)		−0.08 (−0.63 to 0.48)		0.04 (−1.0 to 1.1)		0.16 (−1.1 to 1.4)		0.01 (−0.72 to 0.73)	
61617	−0.98 (−2.3 to 0.38)		−0.08 (−0.39 to 0.22)		−0.26 (−0.84 to 0.31)		−0.25 (−0.94 to 0.44)		−0.15 (−0.54 to 0.25)	
61618	−1.2 (−2.9 to 0.55)		−0.14 (−0.52 to 0.24)		−0.03 (−0.76 to 0.69)		−0.25 (−1.1 to 0.62)		−0.45 (−0.95 to 0.05)	

Abbreviations: CES-D, Center for Epidemiological Studies-Depression; IIEF, International Index of Erectile Function; OGTT, oral glucose tolerance test.

^a^The ratio of T:E2 was found to have superior model fit (using Akaike information criterion) compared to the adjustment for T and E2 separately in these multivariate models.

### Adjusted Effects of Treatment With Testosterone

**Figure 1.**
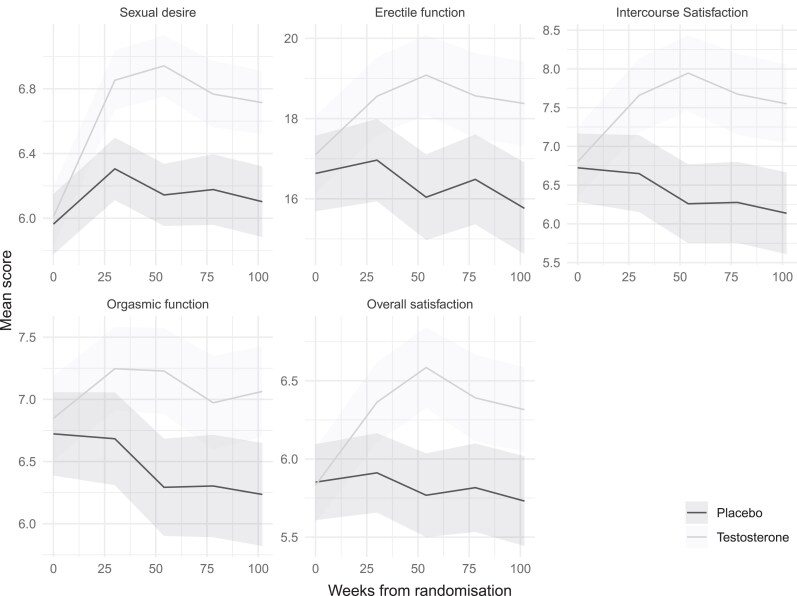
Unadjusted means by treatment over visits for each IIEF domain.

There was an effect of testosterone treatment on each of the 5 individual IIEF-15 domains (Fig. 1), with the largest effects appearing at 54 weeks. However, on assessment of interactions between time and treatment, there was a consistent treatment effect over time for the 5 domains (all *P*-interaction > .08; Supplementary Table S8 (21)). The adjusted means for each domain of sexual function at baseline and 2 years in the testosterone and placebo groups are shown in Table 3. The associations between testosterone treatment and average change in sexual function domains over the 2 years are shown in Table 2. In adjusted models, testosterone treatment was associated with mean increases of 2.5 (95% CI, 1.7-3.4) of 30 in the erectile function, 0.68 (95% CI, 0.49-0.86) of 10 in sexual desire, 0.87 (95% CI, 0.52-1.2) of 10 in orgasmic satisfaction, 1.5 (95% CI, 1.1-1.9) of 15 in intercourse satisfaction, and 0.70 (95% CI, 0.46-0.95) of 10 in the overall satisfaction domains respectively, compared with placebo (*P* < .001 for all). Results were consistent when adjusting for testosterone concentrations and E2 at baseline separately (data not shown).

**Table 3.** Adjusted means from models for each IIEF domain*^a^*

	Baseline means		2-y means	
	Placebo	Testosterone	Placebo	Testosterone
Erectile function	17 (16-18)	17 (16-18)	15.3 (13.8-16.8)	17.9 (16.4-19.3)
Sexual desire	6.0 (5.8-6.2)	6.0 (5.8-6.2)	6.2 (5.9-6.6)	6.9 (6.6-7.2)
Orgasmic function	6.8 (6.5-7.2)	6.8 (6.5-7.2)	6.3 (5.7-6.9)	7.2 (6.6-7.8)
Intercourse satisfaction	6.8 (6.4-7.3)	6.9 (6.4-7.3)	5.7 (4.9-6.5)	7.2 (6.5-7.9)
Overall satisfaction	5.9 (5.6-6.1)	5.9 (5.6-6.1)	5.3 (4.9-5.8)	6.0 (5.6-6.5)

Abbreviations: CES-D, Center for Epidemiological Studies-Depression; IIEF, International Index of Erectile Function; OGTT, oral glucose tolerance test.

^a^Results are averaged over marital status, employment, education, smoker, site, baseline T:E2 ratio, baseline 2 hour OGTT, baseline CES-D score, and baseline domain score.

**Figure 2.**
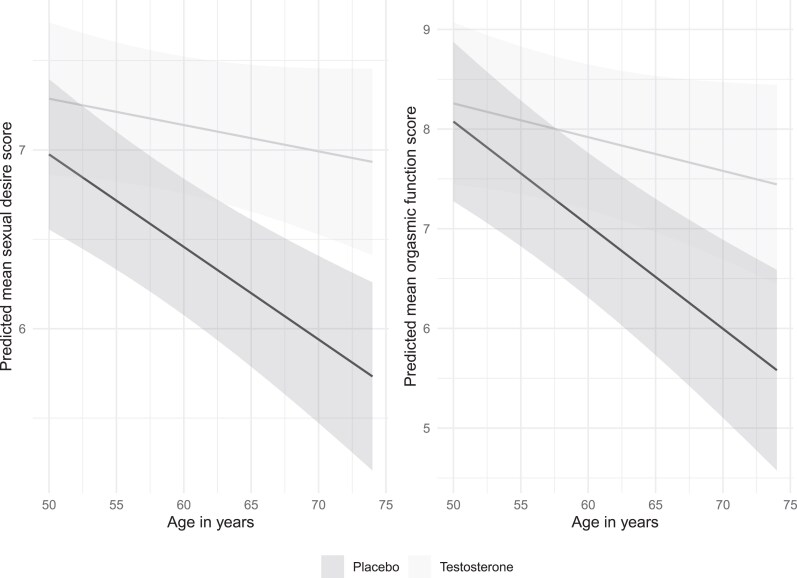
Adjusted mean sexual desire and orgasmic function domains, showing the interaction between age and treatment. Models are adjusted for visit, baseline domain score, and other baseline risk factors.

Interactions between baseline risk factors and treatment were explored for each domain, to ascertain if baseline characteristics predicted differential treatment effects. There was an interaction between baseline age and testosterone treatment, for sexual desire and orgasmic function (both interaction *P* values = .014), with larger treatment effects seen in older men (Fig. 2). No other baseline characteristics (including T < 12 nmol/L vs T ≥ 12 nmol/L) explored predicted differential treatment effects (Supplementary Table S9 (21)) No interactions between treatment and baseline hypertension medication use were found (data not shown). Treatment effects were similar when adjusted for (SSRI and serotonin norepinephrine reuptake inhibitor [SNRI] antidepressants and PDE5-I use (Supplementary Table S10 (21)).

### Accounting for Changes in Risk Factors Over Time

**Figure 3.**
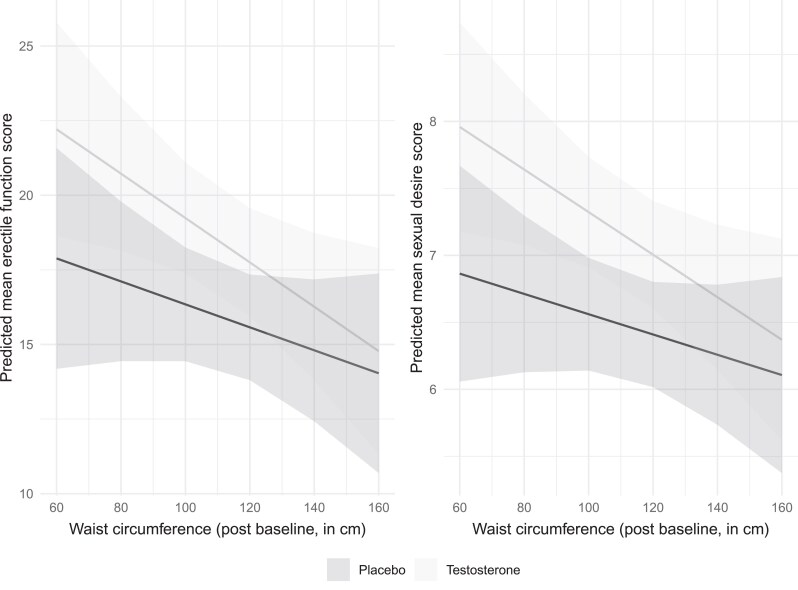
Adjusted mean erectile function and sexual desire domains, showing there is no evidence of an interaction between waist circumference and treatment (both *P* > .23).

Changes in waist circumference over time had associations with the erectile function and sexual desire domains only (Supplementary Table S11 (21)). Increases in waist circumference after baseline were associated with small mean decreases in both the scores on the erectile function (0.06 units per 1 cm) and sexual desire domains (0.01 units per 1 cm). These effects were similar in the placebo and testosterone treatment groups (both interaction *P* values >.23, Fig. 3).

Changes in blood pressure measurements (systolic or diastolic) had no effect on any IIEF-15 domain (Supplementary Tables S12-13 (21)).

**Figure 4.**
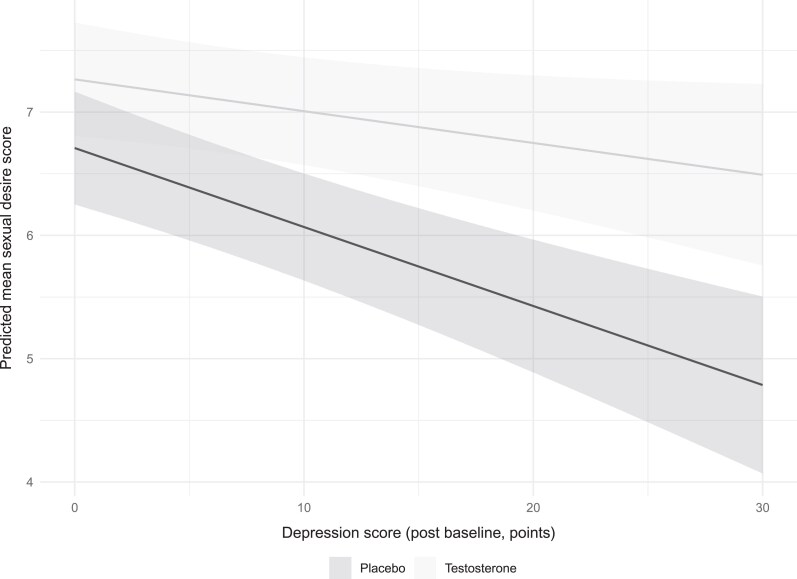
Adjusted mean sexual desire score, after accounting for depression scores on-study and the interaction between depression scores and treatment (*P* = .026).

Changes in CES-D scores were associated with all 5 IIEF-15 domains (Supplementary Table S14 (21)). In all cases, increases in CES-D scores were associated with decreases in the scores for all sexual domains (*P* < .001). These effects were constant over treatment groups for all domains except sexual desire (interaction *P* value = .026, all other *P* > .15). As shown in Fig. 4, those with higher depression scores on the CES-D had a larger treatment effect relative to placebo than those with lower depression scores. In a separate analysis, there was no association between treatment and depression at 2 years (*P* = .95), after adjusting for baseline depression and baseline serum testosterone.

Changes in OGTT were not associated with erectile or sexual domains (both *P* values > .2, data not shown).

### Exploration of Clinically Significant Improvement or Deterioration at 2 Years

For erectile function, the proportion with clinically significant change (clinically significant improvement [CSI] or deterioration) was generally similar between the treatment groups (*P* = .067), with 23% of participants experiencing a CSI in the testosterone group, and 20% in the placebo group. Erectile function deteriorated in 29% and 37% of men in the testosterone and placebo treatment groups, respectively (Table 4). For sexual desire, a greater proportion had CSI (32% vs 22%) and a smaller proportion had deterioration (25% vs 36%) in the testosterone and placebo groups, respectively (*P* < .001).

**Table 4.** Categorization of the changes in erectile function and sexual desire at 2 years, by treatment

	Placebo, N = 395	Testosterone, N = 430	*P* value*^a^*
**Change in erectile function from baseline**			.**066**
Deterioration (<0)	145 (37)	124 (29)	
Improvement (0 to <4)	167 (43)	203 (48)	
Clinically significant improvement (≥4)	79 (20)	95 (23)	
Unknown	4	8	
**Change in sexual desire from baseline**			**<**.**001**
Deterioration (<0)	140 (36)	102 (25)	
Improvement (0 to <2)	163 (42)	181 (44)	
Clinically significant improvement (≥2)	85 (22)	131 (32)	
Unknown	7	16	

^a^Pearson's chi-squared test.

Predictors of CSI for each measure were next explored. For both erectile function and sexual desire, the changes in waist circumference and CES-D scores at 12 months were not predictive for CSI. For erectile function, only baseline erectile function scores and age were predictive of CSI, with lower baseline erectile function scores and those that were younger more likely to have a CSI after adjustment for other factors (*P* < .001 and *P* = .009, respectively; Table 5). For sexual desire, the interaction between age and treatment, and baseline score of sexual desire were predictors of CSI. Those at higher ages had a larger effect of testosterone treatment and a greater chance of having a CSI in sexual desire (*P* = .04; Supplementary Fig. S1 (21)). For example, a 70-year-old patient assigned to placebo has a 10% chance of having a CSI, compared with 27% chance for a 70-year-old patient assigned to testosterone. A 50-year-old patient has a 30% chance of CSI, regardless of treatment assigned, keeping all other covariates constant. Those with lower baseline scores for sexual desire more likely to have a CSI (odds ratio per-unit increase: 0.64; 95% CI, 0.58-0.70; *P* < .001).

**Table 5.** Predictors of clinically significant improvements and deteriorations in sexual desire and erectile function

	Clinically significant improvements of sexual desire				Clinically significant improvements of erectile function			
	Model 1: with change in waist circumference		Model 2: with change in CES-D		Model 3: with change in waist circumference		Model 4: with change in CES-D	
Characteristic	OR (95% CI)	*P* value	OR (95% CI)	*P* value	OR (95% CI)	*P* value	OR (95% CI)	*P*-value
Baseline subscale score	0.64 (0.58-0.70)	<.001	0.64 (0.58-0.70)	<.001	0.94 (0.92-0.96)	<.001	0.94 (0.92-0.96)	<.001
Treatment with testosterone	0.05 (0.00-1.43)	.081	0.05 (0.00-1.52)	.088	1.19 (0.83-1.70)	.35	1.21 (0.85-1.73)	.30
Age at baseline (y)	0.94 (0.90-0.98)	.002	0.94 (0.90-0.98)	.002	0.96 (0.93-0.99)	.008	0.96 (0.93-0.99)	.009
Baseline CES-D score	1.01 (0.97-1.05)	.72	1.01 (0.96-1.05)	.79	1.02 (0.98-1.06)	.34	1.00 (0.96-1.05)	.84
Baseline waist circumference (cm)	1.00 (0.99-1.02)	.64	1.00 (0.99-1.02)	.64	1.00 (0.98-1.01)	.84	1.00 (0.99-1.01)	>.99
Change in waist circumference at 12 mo (cm)	1.00 (0.98-1.03)	.77			1.0 (0.97-1.02)	.70		
Change in CES-D score at 12 mo			0.99 (0.95-1.03)	.64			0.97 (0.93-1.01)	.11
Testosterone*^a^* age (interaction)	1.06 (1.00-1.12)	.034	1.06 (1.00-1.12)	.037				

	Deterioration in sexual desire				Deterioration in erectile function			
	Model 5: with change in waist circumference*^b^*		Model 6: with change in CES-D*^b^*		Model 7: with change in waist circumference		Model 8: with change in CES-D	
Characteristic	OR (95% CI)*^a^*	*P* value	OR (95% CI)*^a^*	*P* value	OR (95% CI)*^a^*	*P* value	OR (95% CI)*^a^*	*P* value
Baseline subscale score	1.44 (1.32-1.57)	<.001	1.43 (1.31-1.57)	<.001	1.03 (1.02-1.05)	<.001	1.03 (1.02-1.05)	<.001
Treatment with testosterone	0.53 (0.38-0.74)	<.001	0.55 (0.39-0.76)	<.001	0.65 (0.48-0.89)	.006	0.65 (0.48-0.88)	.005
Age at baseline (y)	1.05 (1.02-1.07)	<.001	1.05 (1.02-1.08)	<.001	1.04 (1.01-1.07)	.004	1.04 (1.01-1.07)	.004
Baseline CES-D score	0.98 (0.94-1.02)	.31	0.98 (0.94-1.03)	.47	1.05 (1.01-1.08)	.011	1.07 (1.03-1.11)	<.001
Baseline waist circumference (cm)	1.00 (0.98-1.01)	.78	1.00 (0.98-1.01)	.83	1.00 (0.98-1.01)	.62	1.00 (0.98-1.01)	.48
Change in waist circumference at 12 mo (cm)	1.00 (0.97-1.03)	.83			1.01 (0.99-1.04)	.33		
Change in CES-D score at 12 mo			1.01 (0.97-1.05)	.51			1.06 (1.02-1.09)	.001

Abbreviations: CES-D, Center for Epidemiological Studies-Depression; OR, odds ratio.

^*a*^OR (95% CI) odds ratio and 95% confidence interval.

^*b*^Interaction between age and treatment was *P* > .5 for each model and therefore removed from the models.

## Discussion

In men aged 50 to 74 years with waist circumference >94 cm, either impaired glucose tolerance or newly diagnosed type 2 diabetes, and a wide range of serum testosterone concentrations (4-33.1 nmol/L; 115-955 ng/dL) as measured by LCMS at baseline, we have shown: first, baseline sexual function was inversely associated with age and waist circumference but not with sex steroid concentrations. Second, over and above a lifestyle program, and independent of baseline serum testosterone concentration, treatment with testosterone for 2 years improved all domains of sexual function. There were no differential treatment effects in those with T < 12 nmol/L vs T ≥ 12 nmol/L, a threshold proposed for starting testosterone therapy when associated with suggestive hypogonadal symptoms (European Society of Endocrinology 2018).

Decreases in sexual desire and orgasmic function with increasing age were mitigated by treatment with testosterone. Treatment effects were similar when adjusted for the concomitant use of SSRI and SNRI antidepressant and PDE5-I medications. Third, independent of testosterone treatment, smaller waist circumference and lower depression symptom scores over the course of the study were associated with higher sexual function scores across all domains. Finally, in the testosterone compared with the placebo group, clinically significant increases in erectile function and sexual desire occurred in 3% and 10% more men, respectively. Lower age and lower baseline erectile function scores predicted clinically significant increases in erectile function with testosterone treatment. Higher age and lower baseline sexual desire scores predicted a clinically significant increase in sexual desire with testosterone treatment. Despite the lifestyle program and treatment with testosterone, erectile function and sexual desire deteriorated in 25% and 29% of men, respectively.

Our data extend the findings of 2 other large, randomized placebo-controlled trials that examined the effects of daily transdermal gel treatment on sexual function in middle aged and older men. The T-Trials (28) and TRAVERSE sexual function substudy (29) were trials of 12 and 24 months’ duration, respectively. The novelty of our data relates to the unique design of T4DM, using injectable testosterone supervised by study personnel, as the only large testosterone trial allowing partitioning of the effects of testosterone treatment above and beyond those of a lifestyle program. This is especially important because lifestyle programs have significant and independent benefits on sexual function as well as multiple benefits for cardiometabolic and frailty outcomes in the populations studied (30).

At baseline, the inverse association between sexual function in middle aged and older men and increasing age and waist circumference is consistent with previously published data (6). In contrast to the previously reported inverse relationship between depression symptoms and sexual desire (6), we saw a positive relationship between baseline CES-D and sexual desire scores, which, although statistically significant, had a very small effect size. This is likely a spurious finding because, in T4DM, we excluded men with a baseline CES-D score of 16 or more and the mean baseline CESD-score at baseline was low (5.2 [SD 4.9]).

The absence of significant relationships between baseline serum sex steroid concentrations and any domain of sexual function is in contrast to the T-Trials (31), and large cohort studies of middle-aged and older men that showed weak, but statistically significant, associations between baseline serum testosterone concentrations and erectile function (6, 15) and sexual desire (6, 15, 32). The discrepancy most likely reflects the T4DM study population, which was selected for obesity and dysglycemia, where the comorbid conditions overshadow any association between serum testosterone and sexual function (5, 32, 33), and excluded men with pathological hypogonadism where the association between serum testosterone concentration and sexual function is stronger (34, 35). Consistent with this is the observation that for desire and erectile and intercourse satisfaction the inverse association with waist circumference was limited to those with serum testosterone concentrations <12 mmol/L, suggesting that obesity and related comorbidities may result in both the lowered serum testosterone concentration and reduced sexual function. Although serum testosterone concentration and sexual symptoms have been reported to be more closely associated when serum testosterone is at or below 11 nmol/L (5, 34, 36), we found difference in sexual symptoms in those with testosterone concentrations above or below 12 nmol/L.

Over and above the effects of the lifestyle program and independent of baseline testosterone concentration, treatment with testosterone increased, on average, all measures of sexual function, suggesting a pharmacological effect. The peak effect occurred at around 54 weeks and at least statistically, remained stable thereafter until 104 weeks. This differs from the T-Trials where transdermal testosterone gel improved sexual activity during the first 9 months of the study, with the effect waning to insignificance at 12 months (28). A similar waning effect was not seen in TRAVERSE where transdermal gel was also used (29).

The increase in IIEF-15 erectile function domain score with PDE5 inhibitors is on average 6.5 to 8 points (in a scale of 30) (37). In the current study the increase in erectile function score of 2.5 points with testosterone above placebo was modest, but comparable to the T-Trials (2.64 points) that enrolled an older group of men (age ≥ 65 years, mean 72 years) with lower baseline serum testosterone concentrations <275 ng/dL (<9.53 nmol/L) and not selected for obesity and dysglycemia (28). A large individual participant and aggregate data meta-analysis (17 trials of testosterone vs placebo treatment, N = 3380 men, aged 40 years or older [median age 67 years], and with a serum testosterone concentration <12 nmol/L) reported an increase in IIEF Erectile Function Domain score of 2.14 points. Like the current study, there was no relationship between the treatment response and baseline serum testosterone concentrations (38). Similarly, in another study of 211 men, mean age 55.2 years, the improvement in sexual symptoms with testosterone treatment was independent of baseline immunoassayed serum testosterone concentrations over a wide range of values (39).

Using the 2 sexual desire items on the IIEF-15, we found an average increase of 0.7 points (2.1 points if adjusted to match the erectile function scale of the IIEF-15 on a scale of 10) at 2 years in response to testosterone above the effect of placebo treatment. Comparability to the T-Trials (28) and TRAVERSE (29) is limited because they used noncommensurate scales, the Derogatis Inventory of Sexual Function-Men-II (9) and the Hypogonadism Impact of Symptoms Questionnaire (8), respectively. Furthermore, the TRAVERSE sexual function substudy enrolled 1161 men from the main study (mean age 67 years, serum T < 300 nd/dL [10.4 nmol/L]) who complained of low libido, representing 22% of participants (29), a prevalence equivalent to similarly aged men in the general population (40).

For both sexual desire and orgasmic function, a greater effect was seen in older men where treatment with testosterone prevented some deterioration over time. This is in accordance with studies showing preservation of responsiveness to testosterone with aging (2). These data also suggest that that, with increasing age and/or burden of comorbidities, higher serum testosterone concentrations are required to maintain sexual desire and orgasmic function in men but are insufficient to negate the adverse effect of comorbidities on erectile function.

In the T-Trials, sexual desire increased to a greater extent than erectile function (28), and sexual desire but not erectile function increased in TRAVERSE. These observations are consistent with the greater responsiveness of sexual desire to testosterone (2), and the deleterious and dominant effect of cardiovascular disease on erectile function (2). In T4DM, the magnitude of the average testosterone treatment effect on erectile function was slightly greater than for sexual desire. This may reflect the different scales used, or the differential effects of the lifestyle program alone where on average erectile function decreased slightly and sexual desire increased. When clinically significant improvements were considered, treatment with testosterone resulted in greater proportion of men with improvements in sexual desire (10%) than erectile function (3%). CSI for both erectile function and sexual desire were more likely to occur in men with lower baseline scores. Younger men (lower burden of comorbidities) were more likely to have a clinically significant improvement in erectile function and older men (presumably greater dependence on testosterone) for sexual desire.

A unique feature of T4DM is the lifestyle component. A decrease in waist circumference improved sexual desire and erectile function similarly in both treatment groups but not to a clinically significant extent. A decrease in waist circumference, reflecting a decrease in visceral and to some extent subcutaneous fat mass, achieved by lifestyle change, has been previously shown to improve both sexual desire (12, 41) and erectile function in men (11, 12, 41), including older men who were overweight and had type 2 diabetes (42). In these studies, the magnitude of weight loss was 10% or greater resulting in an increase in IIEF ranging from 1.5 to 3.4 points with a higher magnitude of effect being seen in studies where participants were younger and had uncomplicated obesity, and the largest effect occurred when an exercise program was included.

In our study, a decrease in CES-D score during the study most likely because of a decrease in weight (43) was associated with improved sexual desire and erectile function in both the testosterone and placebo treatment groups. An independent inverse association between depression symptoms and sexual function has been consistently described (44). Independent of change in weight, increased physical activity and improvements in nutrient pattern have both been shown to improve mood (45, 46) and sexual function (47, 48). At the end of the T4DM trial, ∼70% of men in both the treatment and placebo groups were meeting the daily recommended physical activity guidelines as opposed to ∼59% at baseline (14).

The absence of an effect of treatment with testosterone on CES-D score is consistent with our previously reported T4DM study data (14, 43) and in accordance with the T-Trials, which also did not find a significant improvement in mood with testosterone treatment compared with placebo (28). Further, in the large individual participant data meta-analysis referred to previously, compared with placebo, treatment with testosterone did not significantly improve psychological symptoms measured by the Beck Depression Inventory (38). In TRAVERSE, testosterone treatment had a statistically significant but very small effect to elevate mood and increase energy in men with mild to moderate depressive symptoms (Patient Health Questionnaire-9 4-<14), less than what would be expected from an anti-depressant and there was no benefit seen for men with a Patient Health Questionnaire -9 ≥ 15 (49, 50)

Study strengths and limitations.

This is the first large randomized controlled trial to examine the combined effects of a lifestyle intervention with testosterone treatment in a population with, or at risk of, type 2 diabetes and a broad range of serum testosterone concentrations. There was careful on-study evaluation of changes in waist circumference and blood pressure and adjustment for use of SSRI and SNRI antidepressants and PDE5-Is. Retention of participants was excellent. Trained staff administered testosterone and placebo injections, resulting in 100% treatment compliance of retained participants. This is also the first large trial to assess the frequency and factors associated with categorical improvement or deterioration in sexual function.

Limitations include that, compared with the entire T4DM cohort, those in this sexual function substudy were slightly older, had lower weight, waist circumference, and burden of depression scores. The observed effects of testosterone treatment may not be generalizable in the absence of a concomitant lifestyle program, and we do not know what the effect of the same formulation and dose of testosterone would be in this population in the absence of the lifestyle program. Serum sex steroid concentrations were measured just before the next injection at 3 months; therefore, we cannot relate overall exposure to testosterone to improvements in sexual function. In the absence of a validated clinically significant increment for the Sexual Desire Domain of the IIEF, we extrapolated from the Erectile Function Domain and a validated increment on the Psychosexual Daily Questionnaire. We did not systematically enquire about PDE5-I use; however, spontaneous reported use (4%) was only slightly less than in TRAVERSE (6%). There were too few men taking PDE5-Is to analyze whether there was any interaction with testosterone treatment.

We did not collect data relating to sexual relationships in the T4DM trial. Libido can exist with or without a sexual relationship and sexual activity can be with a partner or solitary. The IIEF questionnaire, particularly its domains on sexual desire and satisfaction, focuses on individual sexual function and libido independent of relationship status. Libido, as measured by the Psychosexual Daily Questionnaire, also reflects intrinsic sexual desire. Therefore, while relationship status could provide additional context, we do not feel it limits the assessment of testosterone's effect on libido in this study.

In conclusion, the unique study design of T4DM combining a lifestyle program with a randomized placebo-controlled trial of testosterone treatment allowed us to demonstrate that testosterone treatment improved sexual desire (but less so erectile function) above and beyond the effects of a lifestyle program. Interestingly, testosterone effects were more pronounced in older men, men with higher baseline waist circumference, and those with more depressive symptoms during the trial. A decrease in waist circumference and depression symptoms during the trial improved sexual function independent of testosterone treatment, emphasizing the importance of a holistic treatment approach to sexual dysfunction in older men with central obesity.

## Data Availability

The datasets generated during and/or analyzed during the current study are not publicly available but are available from the corresponding author on reasonable request.

## Abbreviations

CES-D: Center for Epidemiological Studies-Depression
CSI: clinically significant improvement
IIEF: International Index of Erectile Function
LCMS: liquid chromatography mass spectrometry
OGTT: oral glucose tolerance test
PDE5-I: phosphodiesterase type 5 inhibitor
SNRI: serotonin norepinephrine reuptake inhibitor
SSRI: selective serotonin reuptake inhibitor
T4DM: Testosterone for the Prevention of Type 2 Diabetes
