# Correction to: “Testosterone Treatment and Sexual Function in Men: Secondary Analysis of the T4DM (Testosterone for Diabetes) Trial”

**DOI:** 10.1210/clinem/dgaf205

**Published:** 2025-04-11

In the above-named article by Wittert GA, Robledo KP, Handelsman DJ, Inder WJ, Stuckey BGA, Yeap BB, Bracken K, Allan CA, Jesudason D, Jenkins A, Januszewski AS, and Grossmann M (*J Clin Endo Metab*. 2025; doi: 10.1210/clinem/dgaf060), there were errors in the Results section and Table 1.

In the originally published article, the first three sentences of the Results section read “Of the 1007 participants randomized into T4DM, 931 (92% of 1007) had at least 1 IIEF-15 domain measured on study and were included in these analyses (Supplementary Table S1, Supplementary Fig. S1 (21)). A total of 74% (688/931) had complete data, whereas the remaining 26% were missing at least 1 measurement of the 5 domains over the 4 on study timepoints. Of the 931, 471 (51%) were randomized to testosterone treatment and 461 (49%) to placebo, and were well matched for baseline characteristics, including use of PDE5-Is (Table 1).” The authors state that the total number of participants with complete data was mistakenly reported as 931 and should be corrected to 932. In the corrected article, the three sentences now read “Of the 1007 participants randomized into T4DM, 932 (92% of 1007) had at least 1 IIEF-15 domain measured on study and were included in these analyses (Supplementary Table S1, Supplementary Fig. S1 (21)). A total of 74% (688/932) had complete data, whereas the remaining 26% were missing at least 1 measurement of the 5 domains over the 4 on study timepoints. Of the 932, 471 (51%) were randomized to testosterone treatment and 461 (49%) to placebo, and were well matched for baseline characteristics, including use of PDE5-Is (Table 1).”

In the originally published article, the title of Table 1 read “Baseline characteristics of those included in analyses (n = 931) by treatment.” Because the number of participants was incorrect here as well, “931” has been replaced with “932.” In the corrected article, the table title now reads “Baseline characteristics of those included in analyses (n = 932) by treatment.”

The article has been corrected online.

doi: 10.1210/clinem/dgaf060

